# Gene-Confirmed Primary Aneurysmal Bone Cysts of the Jaws: A Qualitative Systematic Review of Clinical, Radiologic, and Histopathological Features

**DOI:** 10.7759/cureus.82484

**Published:** 2025-04-18

**Authors:** Reshma Venugopal, Soumya Makarla, Sudhakara Muniswammappa, Prashanth Ramachandra, Radhika Bavle, Soumita Bhattacharya

**Affiliations:** 1 Department of Oral and Maxillofacial Pathology, Krishnadevaraya College of Dental Sciences and Hospital, Bangalore, IND

**Keywords:** altered hemodynamics, blown-out mandible, gene mutation, primary abc, primary jaw abc

## Abstract

Aneurysmal bone cyst (ABC) is rarely a clinical diagnosis on first take; hence, data pertaining to primary ABC assists in differentiating lesions from related pathologies. The cases of primary ABCs were managed by curettage even with extensive cortical plate expansion, whereas secondary ABCs were managed according to primary pathology. The present review retrospectively studies the case reports of primary ABCs affecting the jaws, reported from the year 2000 (the time when USP6 to CDH 11 gene translocation was detected) to date, to identify cases that were diagnostically confirmed with gene analysis. Confirmed primary ABCs of jaws as defined by the World Health Organization (WHO) 2020 classification were included after a thorough literature search. In cases where gene analysis was not done, criteria considered to diagnose as primary ABC (clinical-radiological characteristics and histopathological features) were studied. Recurrence rate and prognosis were analyzed. A male predominance was noted with a ratio of 2:1. The age group between 10 and 20 years and the mandibular posterior region, followed by the temporomandibular joint (TMJ), were mainly affected. Around 22 of 33 cases showed both buccal and lingual cortical plate expansion. Blown-out cortices, fluid levels on magnetic resonance imaging (MRI) or contrast computed tomography (CT), altered blood, and the key histopathological feature of large blood-filled spaces surrounded by fibrovascular septa were the main clues for diagnosis. Around 28 of 33 cases showed giant cells, and 14 of 33 cases showed calcifications/ossifications. Recurrence was noted in 6 of 13 cases treated by curettage. A majority of primary ABCs are believed to exhibit gene mutations; however, genetic analysis was infrequently performed in the reviewed cases. Since the gene analysis may not be feasible in all cases, studies on histopathological analysis and immunohistochemical (IHC) markers to find clues for confirmatory diagnosis are in need.

## Introduction and background

Aneurysmal bone cyst (ABC) is a “cystic or multicystic, expansile, osteolytic neoplasm composed of blood-filled spaces separated by fibrous septa containing osteoclast-type giant cells,” as defined by the World Health Organization (WHO) 2017 classification series [1]. The recent WHO 2020 classification of bone tumors has classified ABC as a benign osteoclastic giant cell-rich neoplasm [2,3]. ABCs were previously defined as a reactive lesion until the discovery of translocation of chromosomes 16 and 17: t(16;17)(q22;p13) and deletion at chromosome 16-del(16)(q22) by Panoutsakopoulos et al. in 1999. The translocation allows the highly active osteoblast cadherin 11 (CDH11) promoter gene to influence the ubiquitin protease 6 (USP6) located on chromosome 17p13, also known as the TRE2 or TRE17 oncogene. This results in increased USP6 transcription. USP6 and CDH11 translocations are seen mainly in the spindle cells of ABC. The finding of mutations, aggressive clinical behavior, and recurrence rate on simple curettage directed the lesion to be reclassified as a mesenchymal neoplasm. ABCs have a tendency to transform into osteosarcomas, as reported in a few cases. The finding of the gene mutation is important as it distinguishes primary ABCs from secondary ABCs, which do not express the gene mutation [4-6].

As per Henriques et al., ABCs are of three histopathological types. The first one is the classic, vascular, or conventional type that consists of numerous blood-filled sinuses not lined by endothelial cells, separated by fibrous connective stroma. The stroma consists of immature fibroblasts and giant cells with signs of thrombosis. On gross examination, the tumor demonstrates a blood-filled, spongy appearance. The presence of hemosiderin, calcification, and ossification is variable. Clinically, it presents aggressive behavior with destructive growth, cortical bone perforation, invasion into soft tissues, and excessive bleeding during surgery. The second type is solid. This consists of small vascular spaces surrounded by cellular fibrous connective tissue. Foci of fibromyxoid tissue that calcify, along with osteoblastic differentiation and osteoid formation, are evident. The presence of large blood-filled sinusoidal spaces is rare. Bone expansion and bleeding during surgery are minimal. A dense stroma, scanty sinusoids, few blood vessels and caverns, bone expansion (instead of destruction), and oozing of blood (instead of severe bleeding) upon surgery correlate with the solid type. The third type is “mixed,” which consists of features of both vascular and solid types [7-11].

According to the WHO 2017 series, the cystic variant shows blood-filled spaces separated by fibrous septa containing multinucleated giant cells. The spaces are lined by macrophages and fibroblasts. Woven bone formation can be seen, whereas the solid variant shows cellular areas with few cystic spaces [11,12].

The WHO 2020 describes three main histopathological features, which include cellular components consisting of multinucleated giant cells expressing receptor activator of nuclear kappa beta (RANK) resembling osteoclasts and stromal mononuclear and spindle cells expressing high levels of RANK ligand (RANKL). The second feature is the fibrous component, consisting of collagen fibers and extracellular matrix. The third is an osteoid component, which includes organic bone matrix secreted by osteoblasts. Mitoses are also seen. Cytological atypia should not be present, and necrosis should rarely be seen [3,13,14].

The present review retrospectively studies cases of primary ABCs reported in the literature.

## Review

Methods

A thorough literature search in both the print and electronic media was performed. The search engines used were Google Scholar, Medline, Embase, Web of Science, PubMed, and Cochrane Library. The keywords used were “Primary Jaw Aneurysmal Bone Cyst”, “Primary Jaw Solid Aneurysmal Bone Cyst”, Primary Jaw Cystic Aneurysmal Bone Cyst”, “Primary Jaw Vascular Aneurysmal Bone Cyst”, “Primary Jaw Aneurysmal Bone Cyst”, or “Gene Mutation”. The case reports, case series, and case studies of primary ABCs of the jaws after the year 2000, when the translocation of USP6-CDH11 was detected to date, were included in the review process. This was done to ascertain the criteria for confirmatory diagnosis. Identification of whether gene analysis is always done for confirmatory diagnosis or whether the other criteria were considered for diagnosis was the aim of the review process. Abstracts only, newsletters, primary ABC cases without complete clinical, radiological, or histopathological details, and secondary ABCs were excluded. The results obtained were scrutinized by all the authors separately to minimize the risk of bias. Any repetitions of the cases or disagreements in the process of diagnosis and confirmation of the lesion were carefully dissected by the authors through discussions. The results obtained were tabulated, and the variables were evaluated.

**Figure 1:**
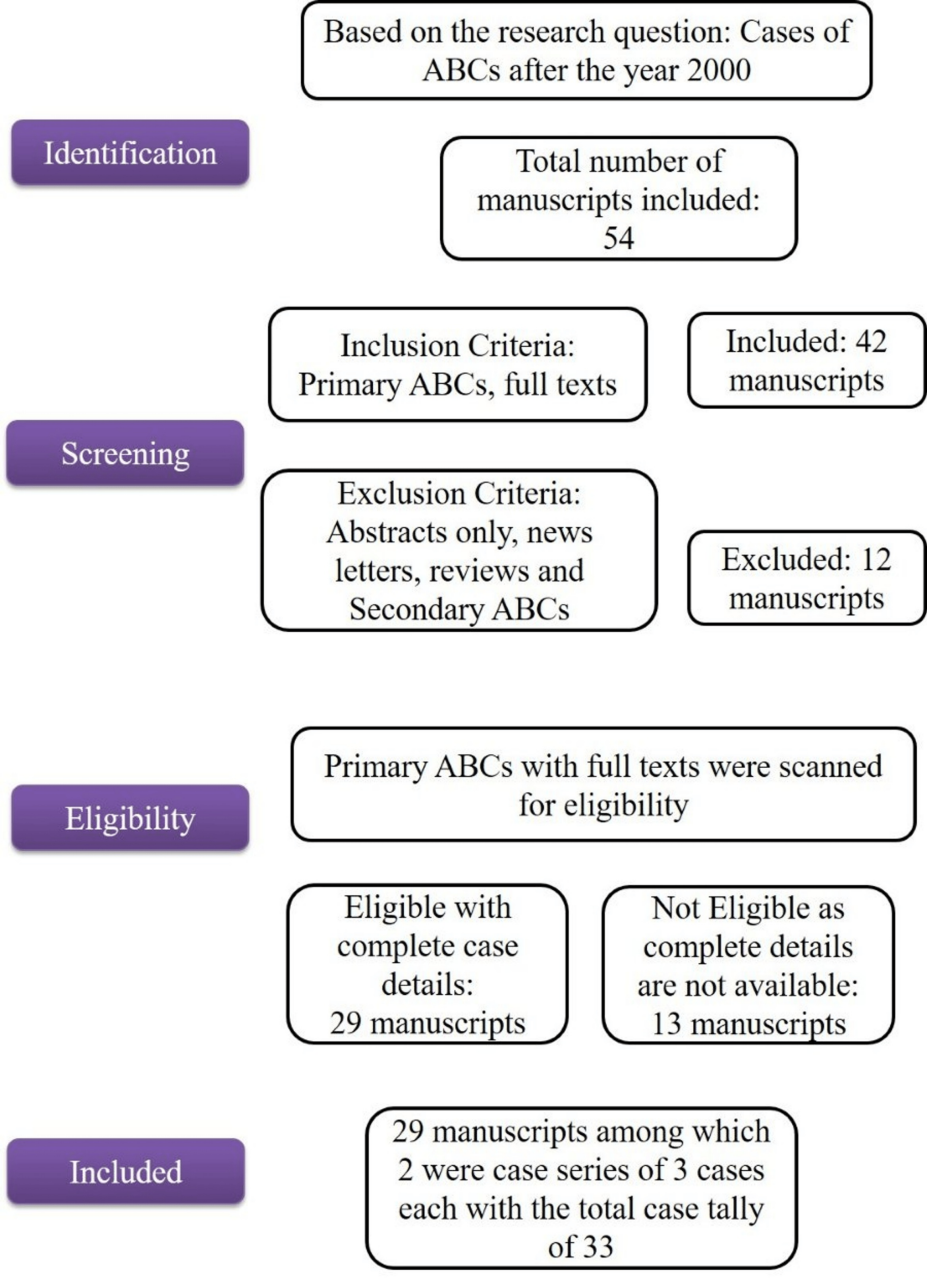
Methodology of selection of cases ABC: aneurysmal bone cyst

On systematic analysis (Figure 1), a total of 33 cases (Table 1) [3,12,15,16-41] were included in the study. The parameters that were analyzed were history of trauma or other cause and demographics (age, gender, site). Clinical presentation such as size/extent of the lesion, associated symptoms, well/ill-defined lesions, and thinning of cortices/cortical perforation. Imaging data such as radiographs, computed tomography (CT), and magnetic resonance imaging (MRI), along with aspiration, surgical, and grossing notes, and histopathological features, were analyzed. In addition, management approaches, recurrence rates, and gene analysis were reviewed. In case gene analysis was not done, the criteria considered to diagnose as primary ABC were noted.

**Table 1:** Details of all the cases searched through Google Scholar, Medline, Embase, Web of Science, PubMed, and Cochrane databases U: unilocular radiolucency; M: multilocular radiolucency; W: well defined; ID: ill-defined; S: swelling; P: pain; E: egg shell crackling; C: curettage; R/E: resection; NR: no recurrence; R: recurrence; CP: cortical perforation; NA: not applicable; LL: lymphocytic leukemia; Max: maxilla; Man: mandible; CS: cellular stroma; Per: perforation; FL: fluid level; B: bleeding; AB: altered blood; SLT: sponge-like tissue; HS: high signal; LS: low signal; TC: thin cortex; AP: anterioposterior; B: buccal expansion; L: lingual expansion; RR: root resorption; RO: reduced opening; F: fracture; Pet: paresthesia

Author and year	Age/gender	History	Site	S1+S2	OPG	CT	MRI	Aspiration	Surgical/grossing	Solid/cystic/mixed	Blood-filled spaces separated by fibrous septa	Giant cells	Woven bone	Gene analysis	Treatment	Recurrence
Gadre and Zubairy 2000 [15]	12/F	Trauma 18 M	Left TMJ	S+P+E	W, U, B+L	NA	FL	NA	B+SLT	Cystic	P	P	NA	NA	R/E	5Y NR
Motamedi 2002 [12]	18/M	NA	Right TMJ	S+P	ID, M, B+L	CP	NA	Altered blood	B+SLT	Cystic	p	p	p	NA	C - 3 times	3 times
Kiattavorncharoen et al. 2003 [16]	6/M	NA	Right angle of man	S	ID, U, B+L	B+L	NA	NA	NA	Cystic	p	p	p	NA	C	1Y R
Perrotti et al. 2004 [17]	12/M	T cell LL- 7 years back	Left man angle	S	W, U, B+L	B+L	NA	NA	NA	Solid	CS+P	P	NA	NA	RE	5Y NR
Rapidis et al. 2004 [18]	16/M	NA	Left TMJ+ramus+infratemporal	S	W, U, B+L	L per	NA	NA	NA	Mixed	CS+P	p	NA	NA	1 & 2nd - C; 3rd- calcitonin+ R/E	3rd visit-NR-9 M
Morita et al. 2005 [19]	10/M	NA	Right man molar	S	ID, U, B+L	NA	HS blood filled	NA	TC	Cystic	P	P	NA	NA	C	4Y NR
Goyal et al. 2006 [20]	17/M	NA	Left coronoid+temporal+infratemporal	S+P	W, U	TC+Heterogenous	NA	Altered blood	Thin walls+brown fluid	Cystic	P	P	NA	NA	R/E	NA
Fyrmpas et al. 2006 [21]	12/F	NA	Max sinus	S+ diplopia	W, M, AP	Heterogenous	M+large cyst	NA	NA	Mixed	CS+P	P	P	NA	R/E	9M NR
Roychoudhury et al. 2009 [22]	15/F	NA	Left man angle	S	W, M, B+L+ RR	U	NA	NA	NA	Cystic	P	P	NA	NA	R/E	3.4Y NR
Roychoudhury et al. 2009 [22]	30/F	NA	Left man body+ramus	S+P	W, M, B+L+ RR	FL+internal septa	NA	NA	NA	Cystic	P	P	NA	NA	R/E	2.8Y NR
Roychoudhury et al. 2009 [22]	14/M	NA	Right man body+symphysis	S	W, M, B+L+RR+lower border	NA	FL+septa	NA	NA	Cystic	P	P	NA	NA	R/E	2Y NR
Ettl et al. 2009 [23]	17/F	NA	Right TMJ	S+P+E	ID, M, B+L+per	Cystic expansion	FL+septa	NA	M+TC	Cystic	P	P	P	NA	1-C; 2-R/E+calcitonin	R after 6M; RE
Pelo et al. 2009 [24]	10/M	NA	Right TMJ	S+RO	W, M, B	M	HS: center; LS: periphery	NA	TC+green fluid+B	Cystic	P	P	NA	NA	R/E	NA
Breuer et al. 2010 [25]	6/M	NA	Right man post	S+P	W, M, B+L	Cystic	M+vascularized	NA	NA	Cystic	P	P	NA	NA	Embolised+R/E	2Y NR
Devi et al. 2011 [26]	25/M	NA	Right man body	S	ID, U, B+L	cystic	NA	Blood	NA	Cystic	P	P	NA	NA	C	NR
Choi et al. 2011 [27]	16/M	Trauma	Right TMJ	S+F	W, M, B	Cystic +F	NA	NA	Blood filled	cystic	P	P	Calcification	NA	R/E	16M NR
Möller et al. 2011 [28]	14/M	NA	Right man molar	S+P+E	W, M, B+L, Per	Cystic	Per	NA	Brown grey tissue filled with blood, no cystic areas	Solid	CS+P	P	NA	NA	C	6M NR
Perumal et al. 2012 [29]	12/M	NA	Left man ramus	S	W, M, B+RR	Enhancing soft tissue mass+septa (152-158 H)	NA	Blood	NA	Mixed	CS+P	P	NA	NA	Embolised+R/E	18M NR
Marchetti et al. 2012 [30]	15/F	NA	Right man angle+body+ramus	S+P+pet	W, U, B, Per	U	NA	NA	Cystic lesion	Cystic	P	P	P	NA	C+R/E after 1 year	5Y NR
Marchetti et al. 2012 [30]	40/M	NA	Left man angle	S+per+pet	ID, M, B	cystic	NA	NA	Lytic+septa+serous	cystic	P	NA	NA	NA	1:C; 2:C	8Y R; 1Y NR
Marchetti et al. 2012 [30]	48/M	NA	Right man body	S+P	ID, M	cystic	NA	NA	Cystic+blood	Cystic	P	NA	NA	NA	C	5Y NR
Zadik et al. 2012 [31]	37/M	NA	Right TMJ	S	ID, U, B+L+per	Cystic+per	NA	Diagnosed as CGCG	NA	Cystic	P	P	NA	NA	R/E	5Y NR
Verma et al. 2013 [32]	8/M	NA	Right max premolar	S+P+eye protrusion	W, M, B+L	M+homogenous mass	T1: hypo; T2: hyper+septa	Altered blood	Cystic-bluish fluid	Cystic	P	P	P	NA	R/E	1Y NR
Ziang et al. 2013 [33]	19/M	NA	Right TMJ	S+P	W, M, B+L_per	cystic+septa	T&T2-HS: center; LS: periphery	NA	TC+brown fluid	Cystic	P	NA	NA	NA	1:C-9M, R/E	6M NR after R/E
Kalia et al. 2014 [34]	19/M	NA	Left man molar	S	W, U, B+L, per	cystic	NA	Blood	Cyst+blood	Cystic	P	NA	NA	NA	R/E	1Y NR
Ariffin and Yunus 2014 [35]	2/M	Trauma 1Y, hereditary thrombocytopenia	Right man body, ramus	S+P+ulcer	ID, M, B	Septa+soft tissue density	T1: heterogenous; T2: M, LS	NA	NA	Cystic	P	P	P	NA	C	1Y NR
Simsek et al. 2014 [36]	23/M	NA	Left man post	S+P	W, U, B+L	Hydraulic periphery	NA	Blood	Pinkish grey friable mass	Cystic	P	P	NA	NA	C	6M NR
Debnath et al. 2016 [37]	8/M	Trauma	Right max anterior	S+P+B	W, M, B+per	TC+septa	Heterogenous mass	Blood	Red soft tissue, firm	cystic	P	P	P	NA	R/E	NR
Gurav et al. 2016 [38]	10/F	NA	right man post	S+P	W, M, B+L	cystic	TC	Blood	No per	Cystic	P	P	P	NA	R/E	NR
Gabric et al. 2017 [39]	11/F	NA	Right man body	S+P	ID, U	ground glass+sclerotic border	NA	NA	NA	Solid	CP+P	NA	P	NA	R/E	NR
Brooks et al. 2019 [40]	27/M	Hypothyroidism	Left TMJ	S+pet	W, M	Heterogenous+Septa+LS fluid	NA	NA	NA	cystic	P	P	P	FISH+Next gen for USP6-CDH 11 mutations	R/E	NR
Álvarez-Martínez et al. 2019 [41]	27/M	Trauma 2Y	Left symphysis	S	ID, M, B+L, RR	Per B&L	NA	Altered blood	Bleeding	Cystic	P	P	P	NA	Embolised+R/E	2Y NR
Shamala et al. 2023 [3]	14/M	NA	Left TMJ+ramus	S+P	W, M, B+L	Heterogenous+septa	NA	NA	Cystic filled with blood	Cystic	P	P	P	NA	R/E	NR

Observations and results

On analysis of 33 cases, primary ABCs of the jaws were more common in males than females (2:1 ratio), and the predominant age group was between 10 and 20 years of age. Five cases were associated with trauma, two cases presented with blood disorders, and one case had a history of hypothyroidism. Most of the cases involved the mandibular posterior region (17 cases), followed by the temporomandibular joint (11 cases) (Table 2).

**Table 2:** Demographic data TMJ: temporomandibular joint

Age				Gender		Site			
<10 years	10-20 years	20-30 years	<30 years	Male	Female	TMJ	Maxillary region	Mandibular anterior	Mandibular posterior
5	20	5	3	22	11	11	3	2	17

All the cases presented with swelling that was associated with pain in 17 cases. Three cases showed paresthesia, and one case reported by Ariffin and Yunus presented with an ulcer (Table 1) [35]. Most of the cases (22) showed both buccal and lingual cortical plate expansion, 11 cases showed ill-defined borders, perforation was seen in seven cases, and root resorption in five cases. Thus, most of the lesions were destructive, multilocular (20 cases), blown-out lesions, though well defined (23 cases), true to their description as aneurysmal (Table 3).

**Table 3:** Clinical and radiological presentation

Swelling +pain	No cortical plate expansion	Buccal plate expansion only	Buccal and lingual cortical plate expansion	Cortical plate expansion with perforation	Root resorption	Well-defined	Ill-defined	Unilocular lesion	Multilocular lesion
17	4	11	22	7	5	23	10	13	20

In most cases, the CT findings confirmed the radiological presentation. Contrast-enhanced CT showed heterogeneous intensity. An MRI scan was performed in 11 cases, which showed both high and low signal intensity. The low signal intensity correlated with fluid levels (Table 1).

Aspiration was done in 11 cases, which showed either altered blood or blood aspirate. Surgical notes were presented in 18 cases; most of the cases (17 cases) were cystic lesions. They presented with blood or altered blood-filled spaces surrounded by thin walls. One case, which was histopathologically diagnosed as solid ABC, presented with greyish-brown tissue. The excised mass was grossly soft, friable, and red to pink in color (reported only in two cases) (Table 1).

On histopathology, three cases each of solid and mixed type were identified. The rest of the 27 cases were cystic in nature. All of the cases presented with areas of hemorrhage surrounded by fibrous connective tissue. About 28 cases showed the presence of giant cells, and 14 cases showed the presence of new bone formation or calcifications. Among the cases that showed bone formation, all three cases of the maxillary region and five TMJ cases were involved. Fluorescent in situ hybridization (FISH) and next-generation sequencing were done to identify the CDH11 to USP6 mutations in one case. Cases that were diagnosed as solid type showed fewer vascular spaces filled with blood/areas of hemorrhage and more cellular stroma. In mixed cases, the large vascular spaces and cellular stroma were in proportion (Table 4).

**Table 4:** Histopathological presentation and treatment ABC: aneurysmal bone cyst

Variants of ABC			Blood-filled spaces surrounded by fibrovascular tissue		Giant cells		Calcification/ossification		Gene analysis		Treatment	
Cystic	Solid	Mixed	Present	Absent	Present	Absent	Present	Absent	Done	Not Done	Curettage	Resection
27	3	3	33	0	28	5	14	19	1	32	13	20

Twenty cases were treated by resection/excision and showed no recurrence. Three cases among these 20 cases were embolized before resection. Among the 13 cases that underwent curettage, six cases recurred and were resected either on the second or third visit (Table 5). Two cases were put on calcitonin as another treatment option. Calcitonin inhibits osteoclastic activity and promotes trabecular bone formation with increased mineral density [42].

**Table 5:** Analysis of recurrence

Treatment			
Curettage - 13 cases		Resection - 20 cases	
Recurrence	No recurrence	Recurrence	No recurrence
6	7	0	20

Discussion

ABCs were first recognized by van Arsdale in 1893 as ossifying hematomas. Jaffe and Lichtenstein were the first to coin the term “aneurysmal bone cyst” for its characteristic blown-out radiographic appearance of the cortex in 1942. Bernier and Bhaskar were the first to describe cranio-skeletal ABCs in 1958. Schajowicz et al., histopathologically, classified ABCs as tumor-like lesions in 1993 [28,38,43-46].

The 2020 WHO classification of bone tumors categorizes ABC as a benign osteoclastic giant cell-rich tumor. The WHO recognizes two types, which are primary, which arises de novo, and secondary, which is associated with an underlying bone lesion. The main difference between primary and secondary ABCs is the age of presentation (between the 1st and 2nd decade in primary cases) and USP6 gene mutation (60-70% of primary cases). Secondary ABCs are seen in the older age group with an underlying pathology, predominantly present with cortical breach, and show no gene mutation. Currently, the WHO 2020 classification considers giant cell lesions of small bones as solid ABCs [2].

The cases of primary ABCs were predominant between 10 and 20 years of age in the mandibular posterior region, as analyzed in other reviews. In the current review, involving mainly the primary ABC of the jaw, male predominance was noticed as against the other reviews, which showed female predominance or no gender predominance. Male predominance correlated with the study done by Motamedi et al. (2014) [2,7,8,10,11,27,34,47].

Most of the cases presented as a swelling, and 17 cases were associated with pain consistent with other case reports and reviews. ABCs showed both buccal and lingual cortical plate expansion and were predominantly multilocular lesions, as concluded in other studies and confirmed through this review [7,8,11].

Enneking has staged ABCs as stage 1, which are static lesions or may heal spontaneously; stage 2 are active lesions with progressive growth without cortical destruction; and stage 3 lesions are locally aggressive with progressive growth and significant cortical destruction [48].

Capanna et al. (1985) divided the ABCs based on the degree of activity as inactive tumors (contained lesion, non-expansile with internal septations, intact cortex, and sclerotic borders), active tumors (slightly symptomatic, expansive with indistinct border, cortical thinning, and a distinct peripheral layer of reactive bone), and aggressive tumors (most active lesions, rapidly expansile, destructive, and extend to surrounding tissues) [13,49].

ABCs are divided into four phases by Dabska and Buraczewski (1969) based on the progression of the lesion as phase 1, or initial small lytic lesions with cortical bone involvement but without lifting of periosteum; phase 2 lesions are rapidly enlarging with a blowout appearance; phase 3 lesions are those that slow or stop after intervention; and phase 4 are those that heal with calcification and ossification [50].

Active or aggressive ABCs with ill-defined borders showed recurrence when curetted. In the present review, three out of 10 cases with ill-defined borders showed recurrence [8,30].

Literature data suggests that fluid levels, low signal of T1 images, and high intensity on T2 MRI are more common features of jaw ABCs than extragnathic ABCs. The fluid levels suggest static blood flow and sedimentation of red blood cells [30]. In the present review, MRI was performed in 10 cases only, and all of them presented heterogeneous intensity with fluid levels. Contrast CT also helps to detect the fluid levels. In the present review, contrast CT showed heterogeneity and fluid levels in six cases.

The manometer test study done by Chari and Reddy has shown that 12 cases out of 17 showed a gradual increase in the blood column when the needle of the manometer was applied to the lesion. The rise of blood oscillates with the pulse. According to Chari and Reddy, these features confirm the diagnosis of ABCs [29,51].

Nevertheless, the cases have to be confirmed histopathologically. This is true in cases of the vascular type of ABC, which presents with blown-out cortices, prompting consideration of malignancy as a clinical differential. Solid ABCs give way too many differentials, and distinction between primary and secondary ABCs can be confirmed only with histopathological examination.

Aspiration was done in 11 cases, which mainly yielded blood or altered blood. Hence, it would be appropriate to always aspirate before proceeding with more invasive procedures.

ABCs are predominantly low-flow lesions on angiograms with rare arterial hypervascularity [29]. This can be confirmed in the present review, as most of the cases on surgery or grossing showed blood or altered blood-filled cystic spaces. Two cases showed increased bleeding during surgery. Such cases were embolized before proceeding with surgery. This correlates with a study done by Motamedi et al., who found that five cases out of 51 (both primary and secondary) showed increased vascularity [11]. Gross details were presented in three cases in the current review, which showed thin cortices and friable red to pink soft tissue mass.

The predominant histopathological features used to diagnose the lesion were spaces filled with red blood cells, surrounded by fibrovascular connective tissue, and the presence of giant cells. According to Jaffe and Liechtenstein, the giant cells seen are mainly involved in the physiological process of bone resorption and may not be a part of the cells forming the tumor [22,44]. Woven bone formation may or may not be seen. As described by Dabska and Buraczewski, formation of bone occurs at the fourth phase, which is the healing phase [13,50]. It has been suggested that the formation of bone indicates the healing phase of ABCs. According to Motamedi et al., mixed types of ABCs were common, as compared to the current review, where cystic or vascular type cases (27 cases) were more common [11]. This correlates with the reviews of Pelo et al. and Vergel De Dios et al. [24,52].

A study done by Urs et al. categorized ABC cases as solid, mixed, or vascular type. This was based on the quantity of stroma, blood vessels, giant cells, and calcified/osteoid material. Accordingly, the solid variant was predominant, followed by mixed and vascular types in their study [53].

Henriques et al. studied nine cases of ABCs, out of which three cases were secondary and six cases were primary [10]. They found solid cases to be more predominant, as against the present study, where cystic type (27 cases) was more predominant.

According to Marchetti et al., the spindle cells in the connective tissue show mitotic activity, but no atypical mitoses were seen. This feature was not mentioned in any of the reviews or the case reports studied in the current review. It has been reported that a mitotic index of seven in extragnathic ABCs shows a higher rate of recurrence [17,30]. Docquier and Delloye compared the histopathological presentation of ABCs with recurrence rate and found that increased cellularity containing stromal and giant cells had a higher recurrence rate [54].

Gene analysis was rarely done to confirm the diagnosis. Gene studies on primary jaw ABCs for confirmation need to be done at any setting that has access to it, as it would affirm the diagnosis. This would also confirm the pathogenesis related to the mutation in the connective tissue cells. This is true in cases of solid ABCs with many differentials.

Recurrence was noted mainly in cases that were curetted (6 of 13 cases), whereas none of the cases that were resected recurred. This suggests resection/excision to be the main treatment option where applicable. According to Zadik et al., the rate of recurrence was higher in the condylar region, mainly due to the inaccessible location that does not allow complete removal of the lesion [31]. Similarly, in the current review, out of six cases that recurred, four were in TMJ.

Various theories to explain the etiopathogenesis of primary ABC

Hillerup and Hjorting-Hansen suggested intramedullary hematoma formation, either due to trauma or fracture, followed by the abnormal reparative process, as a reason for ABC. The hematoma in such cases is connected to a large vessel. This results in altered pressure and ballooning of the lesion [55]. Primary ABCs can be congenital or acquired. Congenital occurs in young individuals without any history of trauma, whereas acquired occurs in the older age group with a history of trauma. In the present review, five cases were associated with trauma, among which the age of the patient was more than 20 years in only one case. This finding does not support the theory that ABCs associated with trauma occur in the older age group. It is also rare to see ABCs associated with large feeding vessels, as per Goyal et al. [11,20,22,26,38].

An alteration in hemodynamics resulting in increased intraosseous venous pressure and expansion of vessel walls is another theory. This was proposed by Jaffe and Liechtenstein. The excessive pressure in the vascular bed is transferred from the arterial side to the venous side, which causes pressure resorption of the bone. Most researchers consider variation in hemodynamics as the main cause of ABCs, as they commonly develop in those areas of the maxillofacial skeleton that possess high venous pressure and marrow content [44].

Primary ABCs can originate from a pre-existing arteriovenous malformation. The primary lesion may be a malformation in the bone, the hemodynamic forces of which result in ABCs. It starts as a microcystic change, which results from venous occlusion or the development of an arteriovenous shunt. This theory gathers some support due to a common operative finding of welling of blood, angiography suggesting a vascular lesion, and the presence of an arteriovenous shunt [12,28,56]. The formation of microcysts containing areas of red blood cells progressively dilates and initiates the formation of vascular spaces. These areas of red blood cells are released from ruptured thin capillaries of the tissue, resulting from stromal collapse [22,56].

Five familial cases of ABCs have been reported in monozygotic twins and siblings, which suggests a familial predilection [43,56].

A few researchers have suggested that ABCs appear to be a cystic version of a central giant cell granuloma or a benign bone tumor of giant cell origin that retains communication with large blood vessels [19,28].

Panoutsakopoulos et al., Oliveira et al., and Dal Cin et al. have reported gene translocation in spindle cells mainly of primary ABCs, which is not seen in secondary ABCs. According to studies, around 60-70% of primary ABCs have shown translocation t(16:17) (q22;p13). This results in fusion of osteoblast cadherin 11 (CDH11) with the entire coding sequence of the ubiquitin protease USP6 gene that is located on chromosome 17p13, also known as TRE17. The TRE17 gene encodes USP6 and the TBC (TRE2-Bub2-Cdc16) domain. The TBC domain encodes guanosine triphosphate (GTP)-activating proteins, mainly small GTPases (like Arf6) that have little-known effects in ABC. The main action is that of USP6 upregulation driven by a highly active CDH11 promoter. TRE17 through USP6 mainly involves the mesenchymal cells [4,5,57,58].

**Figure 2:**
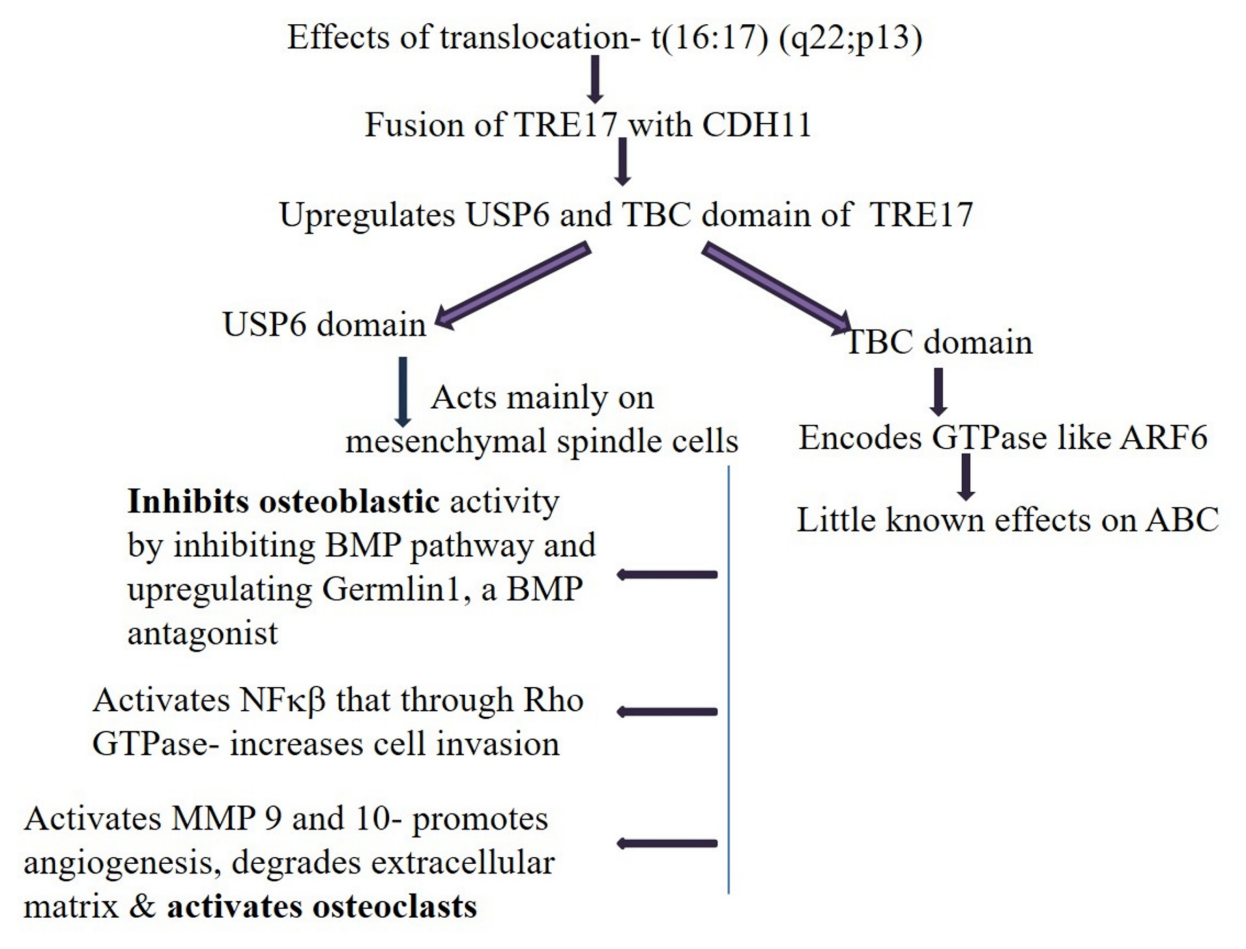
Effects of translocation USP6: ubiquitin protease 6; BMP: bone morphogenetic protein; NF-KB: nuclear factor-kappa beta; GTPase: guanosine triphosphatase; MMP: matrix metalloproteinase; ABC: aneurysmal bone cyst

Due to the translocation, TRE17, through USP6 activity, inhibits osteoblastic differentiation and alters morphology. Multiple regulatory pathways for osteoblastic maturation are affected by TRE17, but the main pathway that is affected is the bone morphogenic protein (BMP) pathway and augmentation of BMP antagonist Gremlin1. It has been observed that exogenous application of BMP4 and use of Gremlin1 inhibitors help the osteoblasts to mature. TRE17 through USP6 activates the canonical nuclear factor-kappa beta (NF-KB) pathway, which is independent of IKB (NF-KB inhibitor) phosphorylation. This regulates plasma membrane endosomal trafficking or actin remodeling via Rho GTPases, which increases cellular invasiveness by increasing cell motility. TRE17 also induces the production of matrix metalloproteinases (MMPs) and inflammatory cytokines, which include granulocyte-monocyte colony-stimulating factor and interleukin-1a. This promotes differentiation or activation of osteoclasts. MMP 9 and 10 promote angiogenesis, inflammation, and extracellular matrix degradation, promoting expansion of the lesion (Figure 2) [8,40,59].

Balance between bone formation by osteoblasts and bone resorption by osteoclasts maintains bone homeostasis. This is disrupted in ABCs as osteoblast maturation is affected. Further, due to activation of the osteoclasts, excessive bone destruction is observed [58].

The expression of TRE17 is seen mainly in normal testes and rarely in other normal tissues. TRE17/USP6 also fuses with other gene partners such as zinc finger 9 (ZnF9), thyroid receptor-associated protein 150 (TRAP150), osteomodulin, collagen 1A1 (COL1A1), and lumican. This generally results in pronounced transcriptional upregulation of the downstream pathway. TRE17 through USP6 also promotes Wnt, JAK1-STAT3, and JUN signaling pathways [40,59].

None of the hypotheses related to pathogenesis individually explains the presentation of ABCs. If we consider the gene mutation studies, the relation of gene mutation to large blood-filled vascular spaces remains unexplained. Also, if the change in hemodynamics hypothesis is considered individually, the changes brought about by gene mutation cannot be explained. Thus, many authors consider pathogenesis related to ABCs as multifactorial with underlying gene mutation [7,56].

Though clinical presentation may vary, detection of gene mutation confirms the diagnosis of primary ABCs in most cases. This is important as the treatment varies between primary and secondary ABCs. It has been reported that extraskeletal ABCs generally do not extend to the end of long bones, but various reports have shown that jaw ABCs can extend and even perforate the cortex. As compared to central giant cell lesions, which most of the ABCs resemble, ABCs mostly occur below the second decade of life, whereas giant cell lesions occur in the mature skeleton. ABCs possess more fibrogenic stroma and large blood-filled spaces than giant cell lesions. As compared to hemangiomas, ABCs lack the smooth muscle constituent [10,12].

Through the present review, we could observe that primary jaw ABCs were more common in men below 20 years of age in the mandibular posterior region, followed by TMJ. They presented with swelling in most cases, which caused both buccal and lingual cortical plate expansion. Thin cystic spaces with fluid levels were seen on MRI. Blood/altered blood was detected on aspiration or during the surgery. The presence of large spaces filled with red blood cells/areas of hemorrhage separated by fibrovascular stroma or myxomatous tissue, and the presence of giant cells were the main histopathological clues to diagnosis.

## Conclusions

ABCs have to be diagnosed by correlating clinical, radiological, surgical, and histopathological presentations. Incisional biopsy would help to rule out the secondary underlying pathology and confirm the diagnosis of primary ABC to go ahead with definitive treatment options. In the current review, trauma was rarely noticed in primary ABCs, which questions trauma as the main etiology for ABCs. Nonetheless, two cases presented with underlying systemic blood dyscrasias, which suggests that these patients have to be evaluated for blood disorders. In cases that cannot be removed thoroughly and aggressive lesions with ill-defined borders, resection should be considered to prevent recurrence. Simple curettage is associated with high recurrence rates, varying from 21% to 50%. Though recurrence has been noted with curettage, their number was less compared to cases that did not recur with curettage (7 of 13 cases) in the present review. These features suggest considering each case on its merit. The area of presentation, extent of involvement, age factor, defined or ill-defined lesions, etc., have to be considered before opting for treatment. Improvement has been shown even with thorough curettage in less active lesions.

Most of the cases or reviews that were reported in the literature focused mainly on clinical, radiological, treatment, and recurrence details, with limited focus on histopathology or further analysis. A study exclusive of histopathology with the focus on the character of the spindle cells, which show the mutation, the character of the giant cells, calcifications, and ossifications, would further give clues for diagnosis as against the differentials. As it has been hypothesized that the TRE17 gene influences osteoblastic maturation, it would also be interesting to see if the stromal spindle cells show osteoblastic lineage. The osteoid tissue formation has been correlated with the healing phase. A study focused on osteoid formation and activity of the lesion would add value to the literature.
